# Directional dependence between major cities in China based on copula regression on air pollution measurements

**DOI:** 10.1371/journal.pone.0213148

**Published:** 2019-03-14

**Authors:** Jong-Min Kim, Namgil Lee, Xingyao Xiao

**Affiliations:** 1 Statistics Discipline, Division of Sciences and Mathematics, University of Minnesota-Morris, Morris, MN, United States of America; 2 Department of Information Statistics, Kangwon National University, Chuncheon, Gangwon, South Korea; 3 Applied Statistics and Psychometrics, Lynch School of Education, Boston College, Chestnut Hill, MA, United States of America; Shandong University of Science and Technology, CHINA

## Abstract

Air pollution is well-known as a major risk to public health, causing various diseases including pulmonary and cardiovascular diseases. As social concern increases, the amount of air pollution data is increasing rapidly. The purpose of this study is to statistically characterize dependence between major cities in China based on a measure of directional dependence estimated from PM2.5 measurements. As a measure of the directional dependence, we propose the so-called copula directional dependence (CDD) using beta regression models. An advantage of the CDD is that it does not rely on strict assumptions of specific probability distributions or linearity. We used hourly PM2.5 measurement data collected at four major cities in China: Beijing, Chengdu, Guangzhou, and Shanghai, from 2013 to 2017. After accounting for autocorrelation in the PM2.5 time series via nonlinear autoregressive models, CDDs between the four cities were estimated to produce directed network structures of statistical dependence. In addition, a statistical method was proposed to test the directionality of dependence between each pair of cities. From the PM2.5 data, we could discover that Chengdu and Guangzhou are the most closely related cities and that the directionality between them has changed once during 2013 to 2017, which implies a major economic or environmental change in these Chinese regions.

## 1 Introduction

Recently, air pollution has become a significant environmental and social problem in China. PM2.5 refers to the concentration of atmospheric fine particulate matter (PM) whose diameter is ≤ 2.5*μ*m. Exposure to PM2.5 is associated with increased mortality rates caused by lung cancer and cardiopulmonary diseases [1–4]. It is generally accepted that PM2.5 is more harmful to human health than PM with diameter > 2.5*μ*m and ≤ 10*μ*m (PM10) [5, 6]. Sources of particulate matter include residential wood burning, coal-fired thermal power generation, agricultural burning, diesel fuel combustion, and natural/industrial dust. PM2.5 can also be generated indirectly when gases and particles interact in the air. China’s air pollution situation is so extreme that ambient air pollution ranked the second highest and household air pollution ranked the third highest in the G20 in terms of disability-adjusted life-years (DALYs) in 2010 [7]. It has been estimated that the air pollution in China contributed to 1.6 million deaths/year in 2014 [8]. In addition to the harmful effects of air pollutants on human health and mortality, pollutants can also have diverse effects on climate and weather due to their complex composition and sources [9–12].

China has regulated ambient air quality since 1982, when it set limits on total suspended particulates (TSP), SO_2_, NO_2_, lead, and Benzopyrene [13]. In February 2012, China adopted a new Ambient Air Quality Standard [14], where limits on PM2.5 were set for the first time. In 2012, major cities in China including the Beijing-Tianjin-Hebei region, Yangtze River delta region, Pearl River delta region, and provincial capitals were required to implement the standards. The current standards were implemented nationwide in 2016; see [15] for more detail.

Nowadays, a large amount of air quality monitoring data is being accumulated, where air pollutants are monitored directly on ground level; see Section 2 for more detail. Due to the increase in the amount of air quality monitoring data in China, numerous studies have been conducted to characterize China’s air quality status and recent trends [8, 16–18]. However, most of the studies focus on analyzing temporal trends of air pollutant concentration and visualizing its spatial distributions at various time scales.

In recent years, there have been efforts to uncover possible sources of air pollution in China [8, 18]. However, it is a challenging task to identify causal effects based on observational data compared to controlled experimental data due to the effects of unobserved variables [19, 20]. On the other hand, under the consideration that a wide variety of factors may influence the PM2.5 level, which may be closely related to environmental and industrial factors, we focus on inferring statistical dependence and causal relations between four major cities in China based on the PM2.5 measurement data as observational evidence. In this study, we included Beijing, Chengdu, Guangzhou, and Shanghai as the target cities in China because they are the four major cities which represent main industrial regions in China.

Approaches to the determination of causal relations based on obserational data include graphical causal modeling [20] such as causal Bayesian networks and structural equation models. Graphical causal models determine directed graph structures satisfying certain conditional independence assumptions called the Markov assumption and faithfulness assumption [19–21]. However, it is well known that a number of directed graph structures cannot be distinguished even if the conditional independence assumptions are satisfied, e.g., *X* → *Y* and *Y* → *X* cannot be distinguished.

Alternatively, the concept of directional dependence has been studied in linear regression settings in the statistics community [22, 23]. Measures of directional dependence using copula regression were investigated in [24]. More generally, regression-based approaches for determining cause and effect variables between two random variables have been suggested in the machine learning community [25–31]. The basic principle is to compare the regression models in alternate directions and investigate asymmetry in the joint distribution.

The purpose of this study is to infer and validate a statistical measure of directional dependence between the selected cities based on time series of PM2.5 concentrations. Copula directional dependence (CDD) is a measure of directional dependences between two regions of interest, which can be applied to non-normal distributions and nonlinear relationships [24, 32, 33]. Contrary to other dependence measures such as correlations and mutual information, the CDD yields bi-directional dependences which measure statistical influence from one region to another.

Copula-based modeling has been widely studied and applied in many fields such as macroeconomics and finance [34, 35] and genetics and biology [36, 37]. Gaussian copula generalized linear models for longitudinal data analysis were introduced in [38]. Multivariate regression analysis using Bayesian inference with Gaussian copulas was proposed subsequently [39]. Later, a framework for joint regression analysis of one-dimensional generalized linear models using Gaussian copulas was proposed [40]. More recently, Gaussian copula marginal regression (GCMR) models were developed [41].

On the other hand, a regression model called the beta regression was proposed [42], where continuous responses are assumed to take values in unit intervals. The beta regression is effective for modeling bounded responses such as rates and proportions due to its flexibility in modeling the shapes and asymmetries of distributions. Various extensions of beta regression models have been proposed [43–46]. For time series data, a beta regression model using the Gaussian copula for bounded time series was proposed in [47], where stationary autoregressive moving average (ARMA) models were adopted for addressing the serial correlation.

In this paper, we apply the CDD proposed in [32, 33]. The suggested CDD measure uses GCMR models [41] and the beta regression model [47]. By using the suggested CDD measure, non-normal and non-linear relationships in the data can be efficiently modeled without any complicated processes of structure search or hyperparameter selection. Moreover, since the CDD is a directional dependence structure, we can further infer directed networks of statistical dependence between the major cities in China.

The rest of this paper is organized as follows. In Section 2, we describe the PM2.5 data and their descriptive statistics. In Section 3, we explain the proposed measure of directional dependence using beta regression. And we describe statistical procedures for processing the time series data and inferring directional dependences between the four cities in China. In Section 4, we present the analyses and results. Discussion and conclusions are given in Section 5.

## 2 Data

### 2.1 Air pollution data

Nowadays, air pollutants are monitored at various locations in many countries. A national real-time air quality monitoring system version 1.0, known as the Air Reporting System, has been operating on the China National Environmental Monitoring Center (CNEMC) website (http://www.cnemc.cn/) since January 2013. The Air Reporting System version 2.0 has been operating since January 2014 and covers 945 sites in 190 cities according to the Ambient Air Quality Standard (GB3095-2012). Other approaches to air quality monitoring include satellite data-based approaches [48–50] and geoscientific modeling [51]. Current air quality monitoring systems such as the Air Reporting System can provide the hourly concentration of various types of air pollutants measured directly at ground level.

Even though the Air Reporting System of China monitors the ambient air quality in a wide range of Chinese regions and cities in real time, most air quality monitoring history is not publicly available. Independently from the Chinese national air quality monitoring system, the US Mission in China started monitoring air quality in 2008 at the US Embassy in Beijing. Since then, monitoring stations have been subsequently established at the US consulates in Shanghai (2011), Guangzhou (2011), Chengdu (2012), and Shenyang (2013) [17]. The air quality status and hourly PM2.5 concentration are available at http://www.stateair.net/.

The PM2.5 time series data analyzed in this study were obtained from the US Mission China air quality website (http://stateair.net). Note that the US Mission China air quality website states that the data are not fully verified or validated; these data are subject to change, error, and correction (http://stateair.net/web/assets/USDOS_AQDataUseStatement.pdf). The historical data files on the stateair.net site can be downloaded manually after agreeing to the data use statement. The obtained data consist of hourly PM2.5 time series of four major Chinese cities, Beijing, Chengdu, Guangzhou, and Shanghai, from January 2013 until June 2017. The original data are hourly measurements of PM2.5 concentration levels measured in *μ*g/m^3^. We removed the data with missing values and then took the daily maximum of the PM2.5 levels.

We note that about 62% of the daily maximum values fell in the night hours (between 21:00 and 4:00) for the 2013 Beijing data. One may consider that air pollution levels during business hours are an indicator of poor health and not values falling in the night hours when the population is asleep. In this sense, it will be very interesting to analyze subsets of the air pollution data measured during business hours for applications to public health in future work. In this paper, on the other hand, we took the daily maximum values to analyze statistical dependence between major cities in China. Since the air pollution level is measured at ground-level, it fluctuates sensitively depending on daily air temperature change. Hence, we remove the effect of daily air temperature change by taking the daily maximum values, which can be indicators of other factors such as coal fuel burning, transportation, industrial activity, and wind.

### 2.2 Descriptive statistics

**Fig 1:**
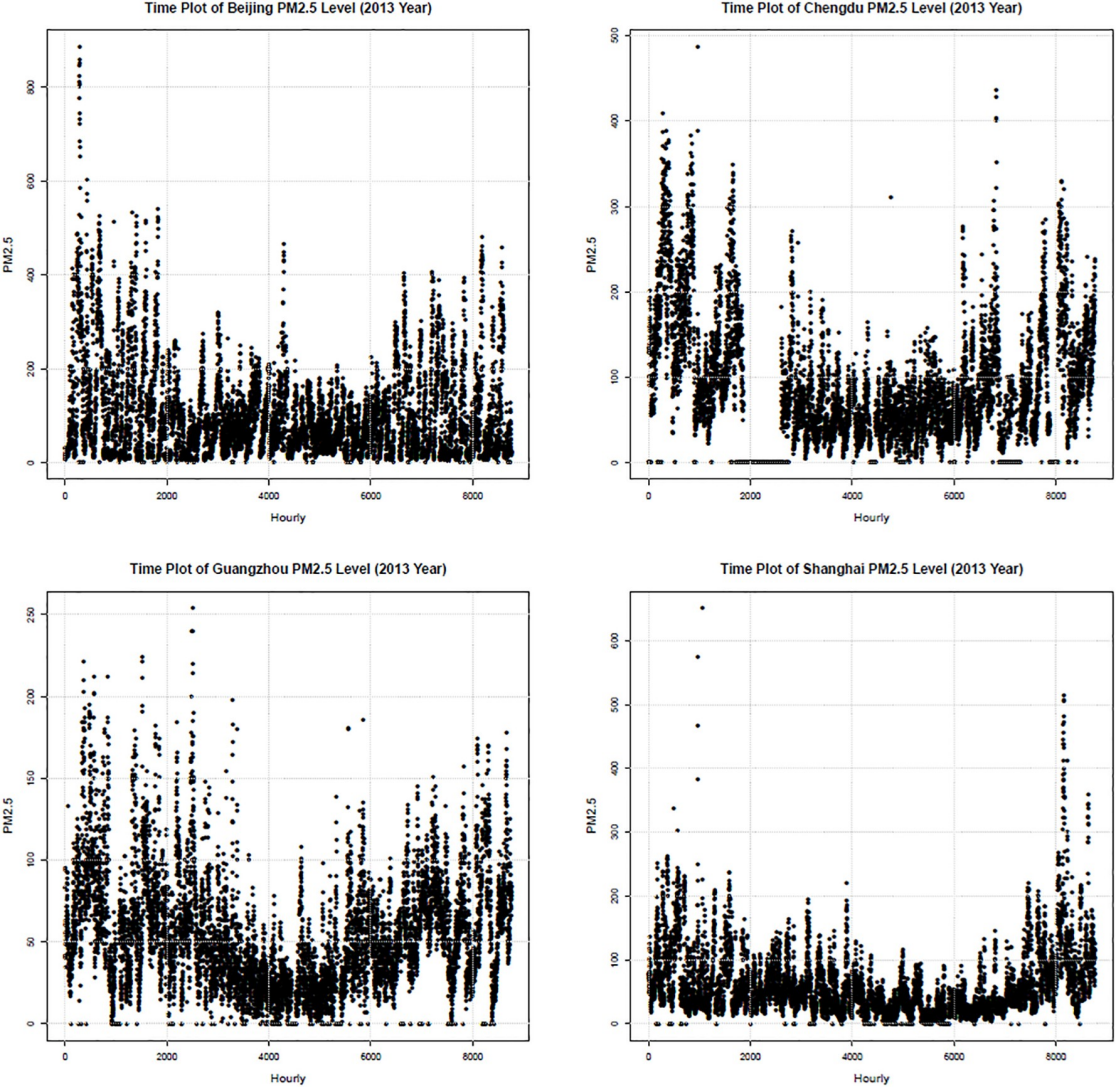
Time series data of PM2.5 levels in the four Chinese cities (Beijing, Chengdu, Guangzhou, and Shanghai) during the year 2013.

Sample time series data of the PM2.5 levels for the year 2013 are illustrated in Fig 1. Hourly measurements of the PM2.5 levels were obtained in *μ*g/m^3^. Missing values were set to zero in the figure. We can see that the PM2.5 levels are relatively high during the winter season (January and December), and Guangzhou and Shanghai show relatively low overall PM2.5 values compared to the other two cities.

**Fig 2:**
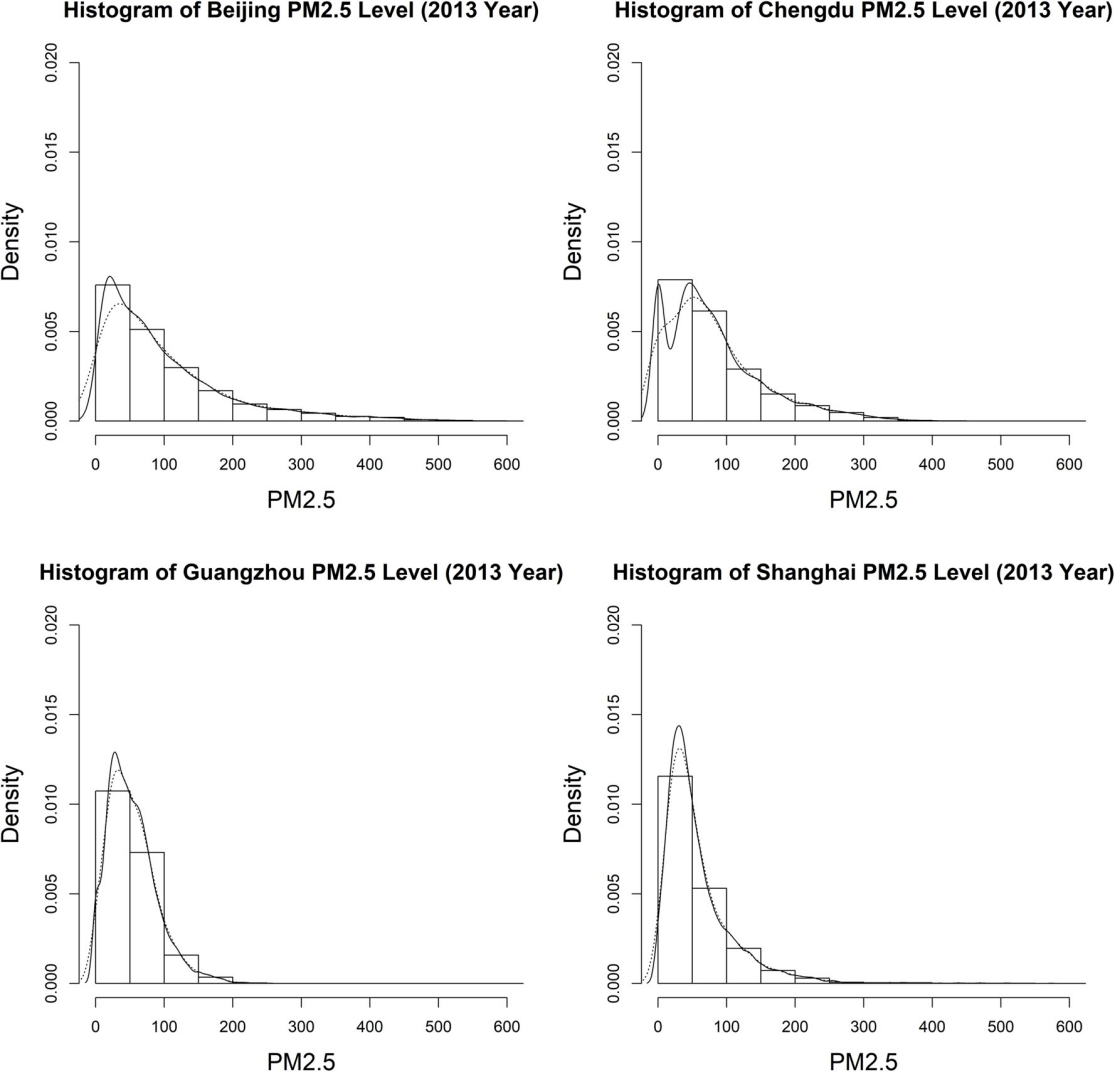
Histograms of PM2.5 levels in the four Chinese cities during the year 2013.

The histograms for the hourly PM2.5 level measurements in the year 2013 are presented in Fig 2. The marginal distribution for each of the Chinese cities is highly skewed and non-normally distributed, which implies that typical statistical dependence measures such as the Pearson correlation coefficient are not suitable.

We have presented summary statistics of the PM2.5 level data for the years 2013 to 2017 in Table 1. During 2013 and 2014, the median value of Beijing was the highest among the four cities. However, it constantly decreased to the second rank in the following years, while Chengdu ranked the first from 2015 to 2017. The median PM2.5 levels of Guangzhou and Shanghai were relatively low and kept decreasing from 2013 to 2016. On the other hand, the maximum of Beijing’s PM2.5 levels was the highest during the years 2013 to 2017 except 2014. The maximum of Shanghai’s PM2.5 levels was the second highest in 2013, but it kept decreasing rapidly and ranked 4th among the four cities by 2016. In summary, the overall PM2.5 levels of the four cities showed a decreasing trend, but each city had its own statistical characteristics.

**Table 1:** Summary statistics of PM2.5 data.

Year		Min.	1st Qu.	Median	Mean	3rd Qu.	Max.	IQR	Skew.	Kurt.
2013	BJ	0	30	71	101	137	886	107	2.00	9.03
	CD	0	33	65	82	114	487	81	1.25	4.63
	GZ	0	27	47	53	73	254	46	1.03	4.49
	SH	0	26	44	59	75	651	49	2.62	15.39
2014	BJ	0	27	71	97	132	671	105	1.79	6.87
	CD	0	43	66	79	101	688	58	1.93	10.66
	GZ	0	20	41	46	64	526	44	1.84	12.53
	SH	0	25	39	49	63	406	38	1.86	8.69
2015	BJ	0	21	53	82	108	722	87	2.28	10.10
	CD	0	38	58	72	90	399	52	1.72	7.15
	GZ	0	18	32	39	50	259	32	1.84	8.08
	SH	0	24	38	49	61	364	37	2.13	9.46
2016	BJ	0	18	49	72	95	782	77	2.27	10.16
	CD	0	40	61	72	95	281	55	1.14	4.28
	GZ	0	13	27	31	42	266	29	1.94	11.39
	SH	0	21	35	44	59	212	38	1.53	5.83
2017	BJ	0	18	42	70	84	684	66	2.85	13.16
	CD	0	39	58	69	85	333	46	1.81	7.48
	GZ	0	18	33	37	52	436	34	2.20	18.55
	SH	0	24	39	45	59	188	35	1.20	4.91

**Fig 3:**
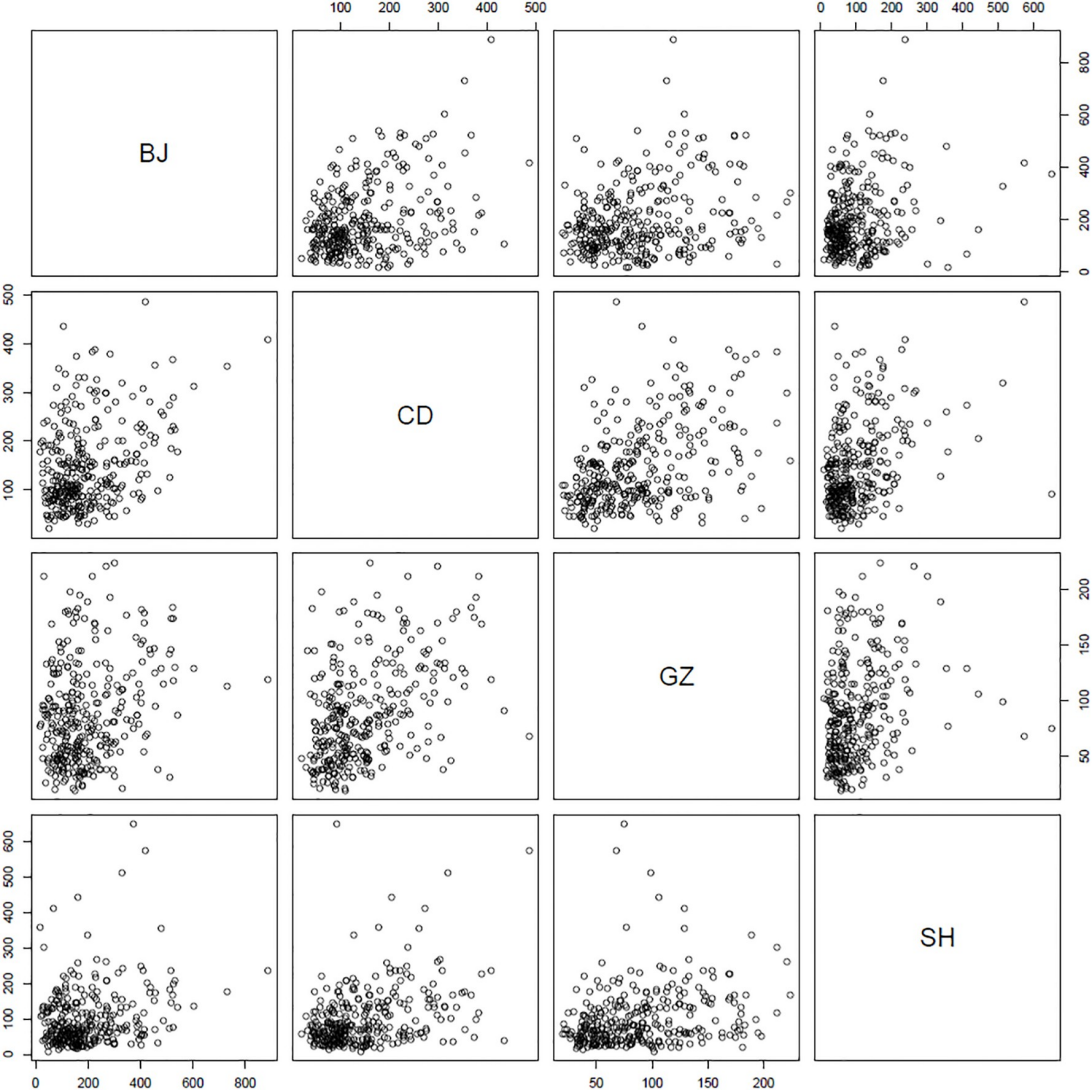
Scatterplot matrix for daily time series data of the PM2.5 levels between the four Chinese cities (BJ: Beijing, CD: Chengdu, GZ: Guangzhou, SH: Shanghai) during the year 2013.

After removing the data with missing values and taking the daily maximum PM2.5 values, we generated scatter plots between each pair of cities, which are illustrated in Fig 3. The data points are distributed in a single cluster, which implies that they were sampled from an identical distribution. In addition, we can find that the distribution is highly skewed, and linear relationship between the variables is not apparent.

As a measure of pairwise correlation between the PM2.5 levels of the four cities, we computed Spearman correlation coefficients, which are summarized in Table 2. Note that the Spearman correlation coefficient is a correlation coefficient between the ranks of the measured values, so it is independent of the skewed marginal distributions.

**Table 2:** Spearman correlation coefficients of daily maximums of the PM2.5 levels between each pair of the four Chinese cities (BJ: Beijing, CD: Chengdu, GZ: Guangzhou, SH: Shanghai) during the years 2013 to 2017.

Year		BJ	CD	GZ	SH
2013	BJ	1	0.27	0.21	0.17
	CD	-	1.00	0.42	0.35
	GZ	-	-	1.00	0.30
	SH	-	-	-	1.00
2014	BJ	1	0.3	0.18	-0.02
	CD	-	1.0	0.25	0.29
	GZ	-	-	1.00	0.23
	SH	-	-	-	1.00
2015	BJ	1	0.36	0.17	0.10
	CD	-	1.00	0.39	0.31
	GZ	-	-	1.00	0.24
	SH	-	-	-	1.00
2016	BJ	1	0.34	0.19	0.13
	CD	-	1.00	0.33	0.33
	GZ	-	-	1.00	0.22
	SH	-	-	-	1.00
2017	BJ	1	0.38	0.32	-0.08
	CD	-	1.00	0.43	0.12
	GZ	-	-	1.00	0.02
	SH	-	-	-	1.00

We can find that

1. Chengdu (CD) had relatively high Spearman correlations with Beijing (BJ) and Guangzhou (GZ) from 2013 to 2017.
2. Correlations between Beijing (BJ), Guangzhou (GZ), and Shanghai (SH) were relatively low from 2013 to 2017.
3. Spearman correlations between Beijing (BJ) and Guangzhou (GZ) slightly increased more recently in 2016 and 2017.

## 3 Methods

A copula is a multivariate joint distribution function, which is an effective approach for describing statistical dependencies within a set of non-normal random variables [52, 53]. Statistical dependence structures can be modeled by choosing a copula function independent of the marginal distributions of each random variable. Since a normal distribution assumption or linearity assumption is not required, copulas can be applied to a wide variety of statistical dependence modeling tasks.

### 3.1 Directional dependence based on copula regression

In this paper, we consider bivariate copulas and suggest a measure of directional dependence between a pair of random variables. A generalization to a measure of directional dependence using multivariate copulas is left for future works, where computationally efficient methods need to be developed.

According to Sklar’s theorem [54, 55], any joint distribution function *F*_*XY*_(*x*, *y*) of two random variables *X* and *Y* can be represented by a bivariate function, *C*(*u*, *v*) composed with the marginal distribution functions, *F*_*X*_(*x*) and *F*_*Y*_(*y*), as

$$\begin{array}{c} F_{XY}\left(x,y\right)=C\left(F_{X}\left(x\right),F_{Y}\left(y\right)\right), \end{array}$$ (1)

where *C*(*x*, *y*) is called the copula. Note that *U* = *F*_*X*_(*X*) and *V* = *F*_*Y*_(*Y*) have a uniform distribution on [0, 1]. We can see that copulas are independent and invariant of marginal distributions. Hence, a copula determines the dependency structure between two random variables independently of any one-to-one continuous transformations of each variable.

Directional dependence refers to asymmetric dependence between random variables. The concept of directional dependence was first investigated under linear regression models in [22, 23]. A copula can be used to define directional dependence in terms of regression [24]. Let *C*(*u*, *v*) denote a copula function, which defines the joint distribution of two random variables (*U*, *V*) whose marginal distributions have a uniform distribution on [0, 1]. Let *C*_*u*_(*v*) be defined by the conditional distribution of *V* given *U* = *u* as

$$\begin{array}{c} C_{u}\left(v\right)\equiv P\left(V\leq v|\;U=u\right)=\frac{\partial C(u,v)}{\partial u}. \end{array}$$

The conditional expectation of *V* given *U* = *u* can be expressed by the copula as [24]

$$\begin{array}{c} r_{V|U}\left(u\right)\equiv E\left[V|\;U=u\right]=1-\int_{0}^{1}C_{u}\left(v\right)\text{d}v. \end{array}$$ (2)

Note that the conditional expectation function *C*_*u*_(*v*) is the regression function of *V* on *U*.

In [24], the directional dependence from *U* to *V* is defined, based on the copula regression function of *V*, by

$$\begin{array}{c} \rho_{U\to V}^{2}\equiv \frac{\text{Var}(r_{V|U}\left(U\right))}{\text{Var}(V)}=\frac{E[{\left(r_{V|U}\left(U\right)-0.5\right)}^{2}]}{1/12}=12E\left[{\left(r_{V|U}\left(U\right)\right)}^{2}\right]-3. \end{array}$$ (3)

Note that the copula directional dependence (CDD) in Eq (3) is the proportion of the variance of *V* which has been explained by the copula regression function *r*_*V*|*U*_(*u*). In the same way, the directional dependence from *V* to *U* is defined by the proportion of the variance of *U* which has been explained by the copula regression function *r*_*U*|*V*_(*v*).

Moreover, note that the CDD defined in Eq (3) is a version of the Spearman’s correlation coefficient. The Spearman’s correlation coefficient can also be expressed in terms of a copula irrespectively of the marginal distributions. The Spearman’s correlation coefficient, *ρ*_*S*_, is the ordinary (Pearson) correlation between *U* = *F*_*X*_(*X*) and *V* = *F*_*Y*_(*Y*). It is well known that *ρ*_*S*_ can be expressed in the following form [56–58]:

$$\begin{array}{c} \begin{array}{cc} \rho_{S} & =12\int \int F_{XY}\left(x,y\right)\text{d}F_{X}\left(x\right)\text{d}F_{Y}\left(y\right)-3\\ & =12\int_{0}^{1}\int_{0}^{1}\left(C\left(u,v\right)-uv\right)\text{d}u\text{d}v. \end{array} \end{array}$$ (4)

Note that if *U* and *V* are statistically independent, then *C*(*u*, *v*) = *uv* on 0 ≤ *u*, *v* ≤ 1, which leads to *r*_*V*|*U*_(*u*) = *r*_*U*|*V*_(*v*) = 0.5. This implies that the directional dependence in Eq (3) and the Spearman’s *ρ*_*S*_ in Eq (4) are measures of the deviations from the joint probability distribution function for indenpendent random variables. See, e.g., [57] for relations between Spearman’s *ρ*_*S*_ and Kendall’s *τ*.

Moreover, for a pair of random variables *U* and *V*, we can estimate and compare the two CDDs, $\rho_{U\to V}^{2}$ and $\rho_{V\to U}^{2}$, to find which copula regression provides a better fit to the data and better explains the variance of the response variable.

### 3.2 Gaussian copula beta regression

The definition of CDD in Eq (3) between two uniformly distributed random variables *U* and *V* can be extended to a pair of continuous random variables *X* and *Y* by defining *U* = *F*_*X*_(*X*) and *V* = *F*_*Y*_(*Y*) and

$$\begin{array}{c} \rho_{X\to Y}^{2}\equiv \rho_{U\to V}^{2}. \end{array}$$ (5)

On the other hand, for estimating the CDD, it is necessary to determine a parametric form of the copula regression function, *r*_*V*|*U*_(*u*). Note that both *U* = *F*_*X*_(*X*) and *V* = *F*_*Y*_(*Y*) are uniformly distributed and take their values in the unit interval [0, 1]. Since *r*_*V*|*U*_(*u*) is a conditional expectation of *V* given *U* = *u*, both the response variable and predictor variable have bounded ranges.

In beta regression, the conditional distribution of a response variable *V* given *U* = *u*_*t*_ is expressed by a beta distribution, Beta(*μ*_*t*_, *κ*_*t*_), with the mean 0 < *μ*_*t*_ < 1 and the precision *κ*_*t*_ > 0 [42]. A beta distribution can flexibly represent a wide range of continuous probabilty distributions defined on the unit interval, that is, various shapes and asymmetries of distributions can be expressed by a beta distribution. The probability density function of *V* given *U* = *u*_*t*_ can be written as

$$\begin{array}{c} f\left(v_{t}|\;\mu_{t},\kappa_{t}\right)=\frac{\Gamma (\kappa_{t})}{\Gamma \left(\mu_{t}\kappa_{t}\right)\Gamma \left(\left(1-\mu_{t}\right)\kappa_{t}\right)}v_{t}^{\mu_{t}\kappa_{t}-1}{\left(1-v_{t}\right)}^{\left(1-\mu_{t}\right)\kappa_{t}-1}, \end{array}$$ (6)

where Γ(⋅) is the gamma function. The mean parameter *μ*_*t*_ is a function of the covariate *u*_*t*_, through the logit function as

$$\begin{array}{c} \text{logit}\left(\mu_{t}\right)=u_{t}\beta_{1}+\beta_{0}. \end{array}$$ (7)

The parameters *β*_0_ and *β*_1_ can be efficiently estimated based on the maximum likelihood approach in the Gaussian copula marginal regression (GCMR) [41, 47]. In the literatures about copula directional dependences, it has been known that the Gaussian copula regression [39] and GCMR [41] are computationally efficient approaches compared to previously known copula directional dependence measures using a family of asymmetric copulas [37, 59, 60]. In the GCMR [41], the cumulative distribution function, *F*(*v*_*t*_|*μ*_*t*_, *κ*_*t*_), for the beta distribution in Eq 6 is used to transform the response variable *v*_*t*_ into *w*_*t*_ = *F*(*v*_*t*_|*μ*_*t*_, *κ*_*t*_). Then, the transformed variable is related with a standard normal random variable *ϵ*_*t*_ by the inverse of the probability integral transform

$$\begin{array}{c} \Psi^{-1}\left(F\left(v_{t}|\mu_{t},\kappa_{t}\right)\right)=\epsilon_{t}, \end{array}$$ (8)

where Ψ is the cumulative distribution function of the standard normal distribution [47]. The above relationship between the responses *v*_*t*_ and the normal random variables *ϵ*_*t*_ is used to formulate the sampling distribution and the likelihood function. See [32, 33, 47] for more details.

### 3.3 Neural network autoregression

In Fig 1, we can see that the air pollution data are far from white noise processes, while their mean and variance change dramatically over time. In order to guarantee a feasible maximum likelihood inference for the beta regression, the serial dependence in the time series data should be removed via a proper modeling of the data [61].

In [32, 33], financial time series data showing conditional heteroscedasticity and serial dependence were preprocessed by employing the asymmetric GARCH(p,q) model [62], and then standardized residuals were generated. In [61], it is noted that GARCH models are employed frequently to remove serial dependence when dealing with financial log-return time series.

In the case of meteorological time series data, nonlinear time series models have been widely applied for addressing irregular or chaotic behavior in the observations [63]. Nonlinear time series models consider that irregularity in the observations can be attributed to nonlinear dynamics occuring on a low dimensional chaotic attractor, which can be reconstructed and used to forecast future observations under appropriate conditions [64, 65]. Traditional linear stochastic time series models such as ARIMA models [66] have influenced the forecasting community significantly; however, they cannot capture the nonlinear dynamics underlying real life observations [67]. On the other hand, many useful nonlinear time series models have been proposed [67, 68]. Among them, artificial neural networks (ANNs) such as feedforward neural networks have been suggested as a promising approach for modeling irregular behavior in time series [69–72]. We adopted the neural network approach for estimating nonlinear autocorrelations in the data by using the nnetar function in the R package **forecast** [73].

Let $y_{it}^{\left(s\right)}$ denote the PM2.5 concentration level at a city *i* = 1, 2, 3, 4, day *t* = 1, 2, …, *T*_*s*_ and year *s* = 2013, …, 2017. The nonlinear feed-forward neural network model for a lagged time series can be written as

$$\begin{array}{c} y_{it}^{\left(s\right)}=f(y_{i,t-1}^{\left(s\right)},y_{i,t-2}^{\left(s\right)},\ldots ,y_{i,t-L}^{\left(s\right)})+\epsilon_{it}^{\left(s\right)}, \end{array}$$ (9)

where *L* is the lag order and *f* is a neural network with *H* hidden nodes in a single layer. The neural network model with *L* lags and *H* hidden nodes is denoted by **NNAR(*L*,*H*)**. The model parameters *L* and *H* were determined automatically by default values, i.e., *L* by the optimal value according to AIC for a linear AR(*p*) model, and *H* by half of the numbers of input values plus one. In addition, the error process $\epsilon_{it}^{\left(s\right)}$ is assumed to be homoscedastic. See the **forecast** package documentation [73] for default preprocessing procedures and default hyper-parameter values for the nnetar function.

After fitting the neural network model in Eq (9) to the data, the fitted values, ${\hat{y}}_{it}^{\left(s\right)}$, are the predicted values which can be written as

$$\begin{array}{c} \begin{array}{cc} {\hat{y}}_{it}^{\left(s\right)} & =f\left(y_{i,t-1}^{\left(s\right)},y_{i,t-2}^{\left(s\right)},\ldots ,y_{i,t-L}^{\left(s\right)}\right)+\epsilon_{it}^{(s)*},\;t\leq T_{s}+1,\\ {\hat{y}}_{i,T_{s}+2}^{\left(s\right)} & =f\left({\hat{y}}_{i,T_{s}+1}^{\left(s\right)},y_{i,T_{s}}^{\left(s\right)},\ldots ,y_{i,t-L+2}^{\left(s\right)}\right)+\epsilon_{i,T_{s}+2}^{(s)*},\\ & \vdots \end{array} \end{array}$$ (10)

The term $\epsilon_{it}^{(s)*}$ denotes a value randomly drawn from normal distributions, which can be used for obtaining prediction intervals. We have $\epsilon_{it}^{(s)*}=0$ because we do not need predicted values for *t* > *T*_*s*_. The residuals can be written as

$$\begin{array}{c} {\hat{\epsilon }}_{it}^{\left(s\right)}=y_{it}^{\left(s\right)}-{\hat{y}}_{it}^{\left(s\right)},\;L+1\leq t\leq T_{s}. \end{array}$$

Due to the model in Eq (9), the residuals, ${\hat{\epsilon }}_{it}^{\left(s\right)}$, can be considered to be not serially correlated.

### 3.4 Bootstrap confidence interval

Let $\Delta \rho_{U,V}^{2}$ denote the difference, $\Delta \rho_{U,V}^{2}=\rho_{V\to U}^{2}-\rho_{U\to V}^{2}$. It measures the difference of the two directional dependences between the two cities *U* and *V*. If $\Delta \rho_{U,V}^{2}>0$, then it means that city *U* affects the other city *V* more than *V* affects *U*.

For a statistical test of the difference, we use the bootstrap resampling method. We resampled a fixed rate from the data, then computed the CDDs repeatedly. In this way, we can estimate the 95% confidence interval for the difference, i.e.,

$$\begin{array}{c} \Delta \rho_{U,V}^{2}\in \left[\text{LB}\left(\Delta_{U,V}\right),\text{UB}\left(\Delta_{U,V}\right)\right], \end{array}$$ (11)

where LB(Δ_*U*,*V*_) and UB(Δ_*U*,*V*_) are the lower and upper limits of the 95% confidence interval. If the confidence interval does not include zero, then we can reject the null hypothesis that there is no difference between the directional dependences $\rho_{U\to V}^{2}$ and $\rho_{V\to U}^{2}$ with a 95% confidence level.

### 3.5 Statistical properties: A review

The proposed CDD using beta regression was proposed recently in [32, 33]. Statistical properties of the proposed CDD measure using beta regression have been assessed numerically in [32] by using simulated data sets. The experimental setting and the results can be summarized as follows. First, an asymmetric bivariate copula was constructed based on the formula of Durante [59], which is written as

$$\begin{array}{c} C\left(u,v\right)=C_{1}\left(u^{\alpha },v^{\beta }\right)C_{2}\left(u^{1-\alpha },v^{1-\beta }\right), \end{array}$$ (12)

where *C*_1_ and *C*_2_ are two symmetric copulas and *α*, *β* ∈ (0, 1) are asymmetry parameters. *C*_1_ and *C*_2_ were selected as a Frank copula with parameter *θ*_3_ = 5 and a Gumbel copula with parameter *θ*_4_ = 40, respectively [32, 59]. It is challenging to obtain a theoretical CDD value, $\rho_{U\to V}^{2}$, of random variables (*U*, *V*) having the specific distribution *C*(*u*, *v*). Instead, a sample of 8,888 correlated pairs, {(*u*_*i*_, *v*_*i*_)}, was generated, and the CDD value was estimated by $\rho_{U\to V}^{2}=0.6421$ and $\rho_{V\to U}^{2}=0.5937$. In order to assess the accuracy of the proposed CDD measure, the CDD value was estimated from each of the thousand independent subsamples with sample size 1000. Based on the thousand estimated CDD values, ${\hat{\rho }}_{U\to V}^{2}$, the accuracy of the CDD was evaluated via the bias and standard error. In summary, the bias was estimated as $\text{bias}({\hat{\rho }}_{U\to V}^{2})=-0.0014$ and $\text{bias}({\hat{\rho }}_{V\to U}^{2})=-0.0002$, and the standard error was estimated as $\text{SE}({\hat{\rho }}_{U\to V}^{2})=0.0002$ and $\text{SE}({\hat{\rho }}_{V\to U}^{2})=0.0003$.

On the other hand, theoretical properties of the CDD using beta regression are under development. For example, the exact CDD value of a given bivariate copula is not easy to obtain in general. In the same way, given a CDD value, it may be interesting to construct an asymmetric or symmetric copula function in a parametric form.

## 4 Results

We applied the neural network approach for autocorrelation estimation described in Section 3.3. Details on the feedforward neural networks and their hyper-parameters used in this paper are presented in Table 3.

**Table 3:** Details on the feedforward neural networks used for autocorrelation estimation. *L*: the number of input nodes, i.e., the lag order, *H*: the number of nodes in the hidden layer.

Year	2013		2014		2015		2016		2017	
Num. nodes	*L*	*H*	*L*	*H*	*L*	*H*	*L*	*H*	*L*	*H*
BJ	1	1	4	2	4	2	2	2	3	2
CD	4	2	4	2	1	1	15	8	2	2
GZ	7	4	5	3	1	1	1	1	5	3
SH	17	9	8	4	10	6	7	4	1	1

**Fig 4:**
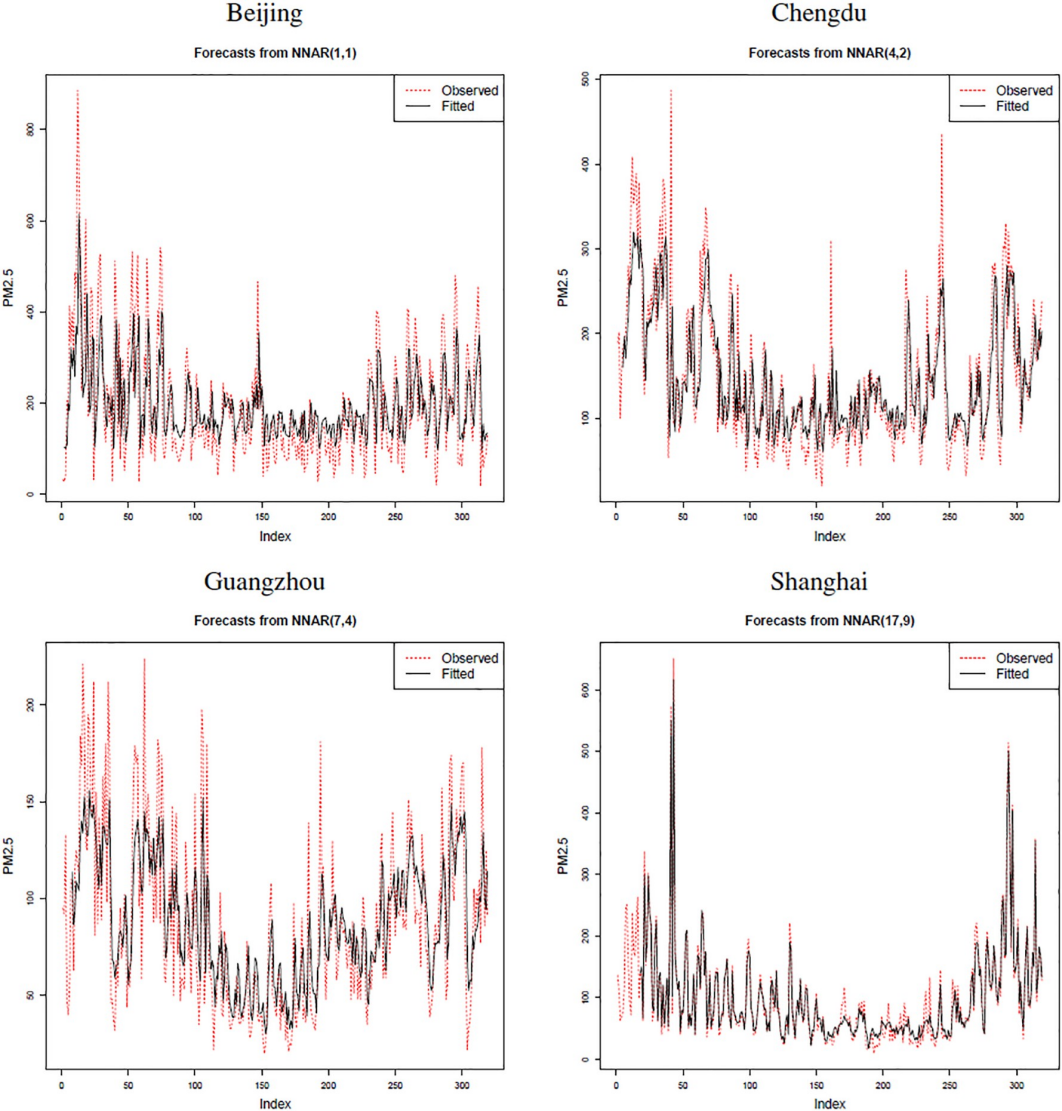
Predicted values (black straight line) of PM2.5 levels of the four Chinese cities during the year 2013 and the observed PM2.5 measurements (red dotted line).

The PM2.5 values predicted by the estimated feedforward neural networks are illustrated in Fig 4, which are for the year 2013. After the prediction, we carried out the Durbin-Watson test for serial correlation based on the residuals. The obtained p-values for the year 2013 were 0.274 (Beijing), 0.686 (Chengdu), 0.275 (Guangzhou), and 0.318 (Shanghai), all of which were larger than the significance level *α* = 0.05. For the other years, we obtained p-values larger than 0.05 as well.

Next, we computed the CDDs between each pair of Chinese cities based on the residuals of the daily PM2.5 levels in each year. The results are summarized in Table 4. Contrary to the correlation coefficients presented in Table 2, the CDDs are not symmetric and are computed in both directions, *U* to *V* and *V* to *U* for each pair (*U*, *V*) of the cities. We remark that the CDD values presented in Table 4 are compared with the square of correlation coefficients rather than correlation coefficients themselves since the definition in Eq (3) is a version of the coefficient of determination in multiple linear regression. In addition, we have computed 95% bootstrap confidence intervals for the difference, $\Delta \rho_{U,V}^{2}=\rho_{V\to U}^{2}-\rho_{U\to V}^{2}$. We can find that most of the confidence intervals do not include zero, which means that the difference $\Delta \rho_{U,V}^{2}$ of directional dependences is significantly different from zero.

**Table 4:** Copula directional dependences (CDDs) between ordered pairs of Chinese cities (BJ: Beijing, CD: Chengdu, GZ: Guangzhou, SH: Shanghai) during the years 2013 to 2017, together with 95% bootstrap confidence intervals (CIs) for the difference of directional dependences. The larger value between the two directional dependences is marked in bold font.

Year		BJ → CD	BJ ← CD	BJ → GZ	BJ ← GZ	BJ → SH	BJ ← SH
2013	CDD	0.0131	**0.0144**	0.0020	**0.0023**	0.0013	**0.0029**
	95% CI	(0.0013, 0.0014)		(0.0002, 0.0002)		(0.0015, 0.0016)	
2014	CDD	**0.0379**	0.0353	0.0053	**0.0113**	**0.0132**	0.0126
	95% CI	(-0.0028, -0.0024)		(0.0059, 0.0061)		(-0.0007, -0.0005)	
2015	CDD	**0.0168**	0.0157	**0.0007**	0.0000	0.0191	**0.0281**
	95% CI	(-0.0014, -0.0011)		(-0.0007, -0.0006)		(0.0087, 0.0091)	
2016	CDD	0.0000	0.0001	**0.0150**	0.0121	**0.0003**	0.0000
	95% CI	(0.0000, 0.0001)		(-0.0030, -0.0027)		(-0.0002, -0.0001)	
2017	CDD	**0.0297**	0.0188	**0.0630**	0.0507	0.0124	**0.0230**
	95% CI	(-0.0110, -0.0107)		(-0.0127, -0.0123)		(0.0106, 0.0108)	
Year		CD→GZ	CD←GZ	CD→SH	CD←SH	GZ→SH	GZ←SH
2013	CDD	0.0117	**0.0176**	0.0050	**0.0053**	0.0025	**0.0026**
	95% CI	(0.0058, 0.0060)		(0.0003, 0.0003)		(0.0002, 0.0002)	
2014	CDD	0.0031	**0.0036**	**0.0215**	0.0149	0.0044	**0.0064**
	95% CI	(0.0006, 0.0007)		(-0.0068, -0.0066)		(0.0018, 0.0020)	
2015	CDD	**0.0277**	0.0182	0.0063	**0.0080**	**0.0130**	0.0091
	95% CI	(-0.0096, -0.0093)		(0.0016, 0.0018)		(-0.0039, -0.0037)	
2016	CDD	**0.0004**	0.0033	0.0000	**0.0004**	0.0060	**0.0137**
	95% CI	(-0.0011, -0.0010)		(0.0003, 0.0004)		(0.0078, 0.0081)	
2017	CDD	**0.0776**	0.0761	**0.0052**	0.0005	0.0031	**0.0047**
	95% CI	(-0.0016, -0.0013)		(-0.0047, -0.0045)		(0.0016, 0.0017)	

**Fig 5:**
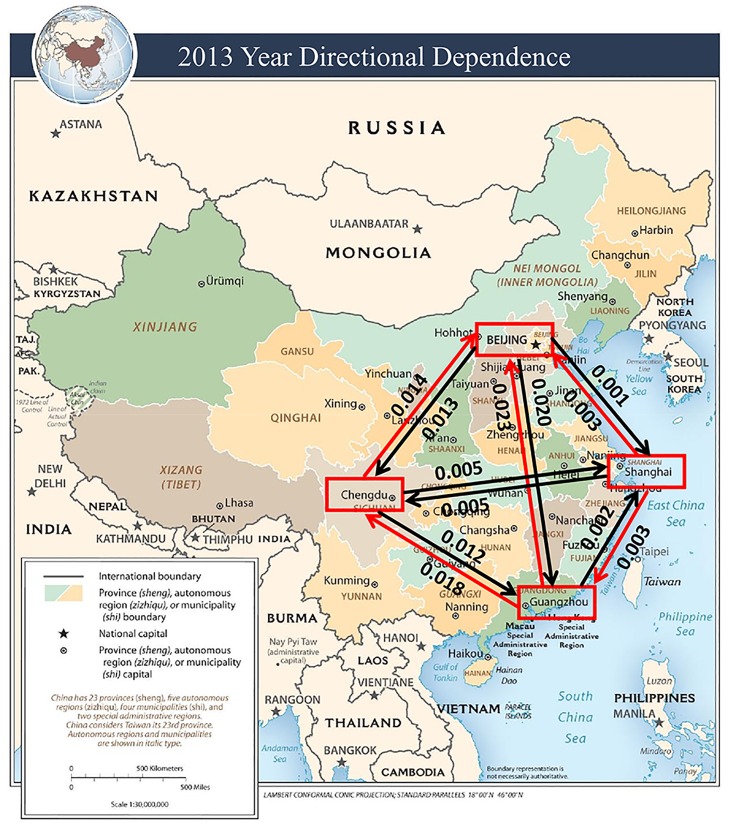
A connectivity map of China based on the directional dependences between the four cities in the year 2013. Base map figure source: CIA Maps (https://www.cia.gov/library/publications/resources/cia-maps-publications/China.html).

**Fig 6:**
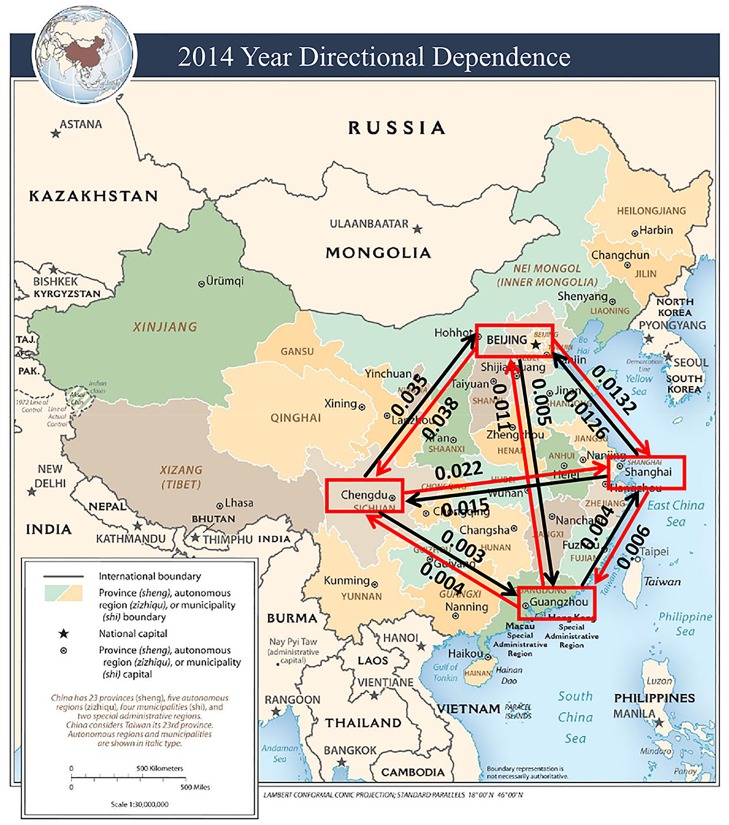
A connectivity map of China based on the directional dependences between the four cities in the year 2014. Base map figure source: CIA Maps (https://www.cia.gov/library/publications/resources/cia-maps-publications/China.html).

**Fig 7:**
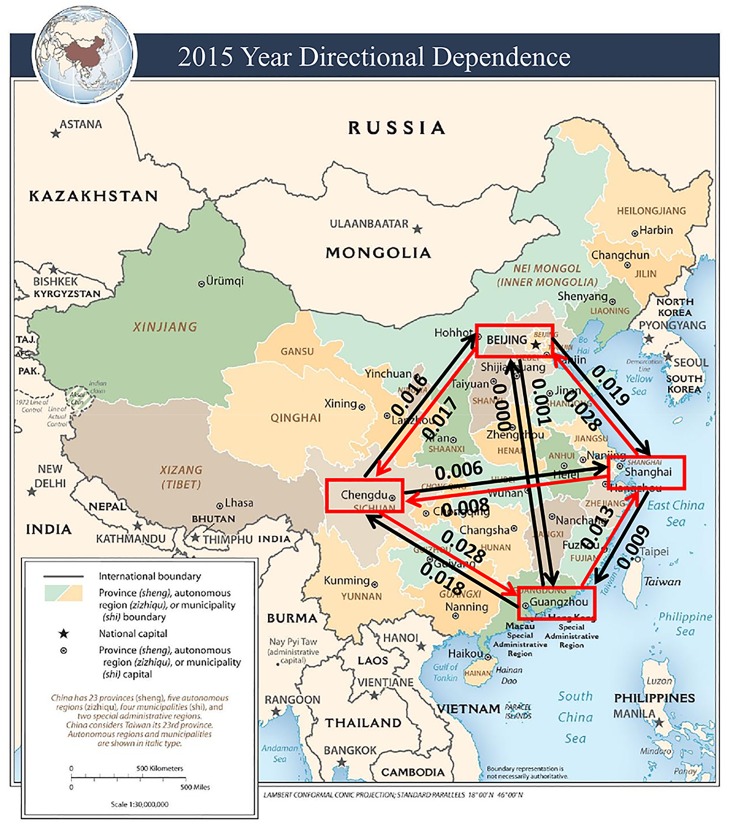
A connectivity map of China based on the directional dependences between the four cities in the year 2015. Base map figure source: CIA Maps (https://www.cia.gov/library/publications/resources/cia-maps-publications/China.html).

**Fig 8:**
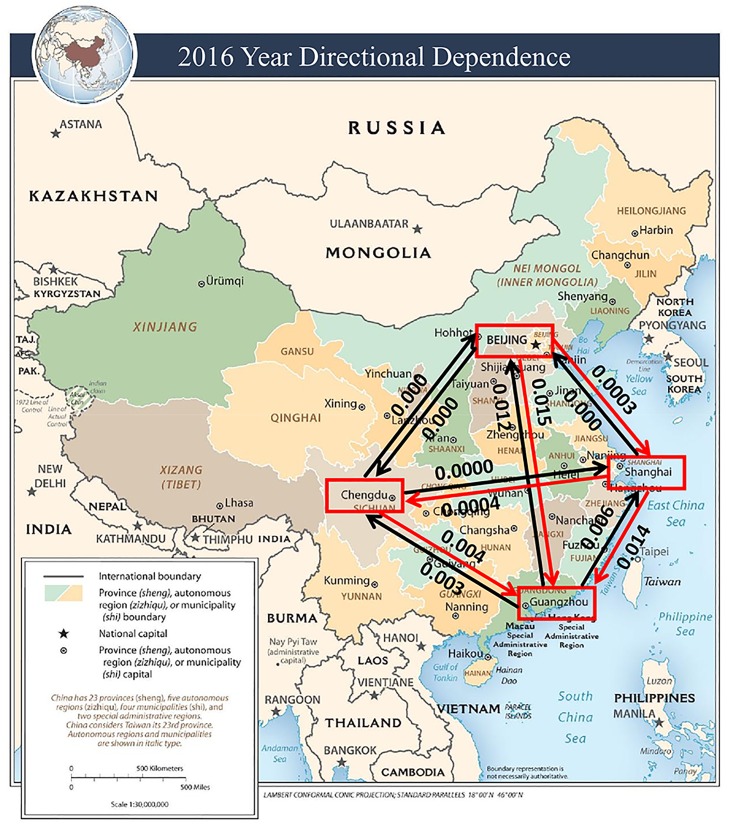
A connectivity map of China based on the directional dependences between the four cities in the year 2016. Base map figure source: CIA Maps (https://www.cia.gov/library/publications/resources/cia-maps-publications/China.html).

**Fig 9:**
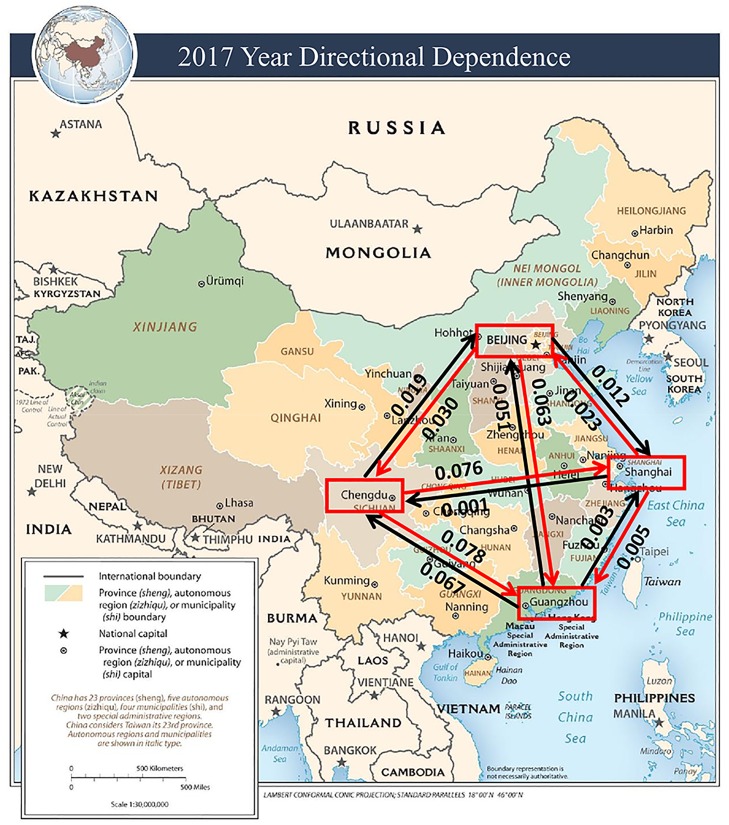
A connectivity map of China based on the directional dependences between the four cities in the year 2017. Base map figure source: CIA Maps (https://www.cia.gov/library/publications/resources/cia-maps-publications/China.html).

The estimated CDD values are visually illustrated in Figs 5 to 9. The labels on the arrows between the four Chinese cities represent the directional dependences between the cities. Several findings from the CDD values are as follows:

1. The CDD between Beijing (BJ) and Chengdu (CD) was relatively high from 2013 to 2017 except in 2016. The CDD from CD to BJ was higher in 2013, but the CDD from BJ to CD became higher in the later years from 2014 to 2017.
2. The CDD between Chengdu (CD) and Guangzhou (GZ) was relatively high in the years 2013, 2015, and 2017. Similarly, the CDD from GZ to CD was higher in 2013 and 2014, but the CDD from CD to GZ became higher in the later years from 2015 to 2017.
3. The CDD between Chengdu (CD) and Shanghai (SH) was relatively high in 2014, in which the CDD from CD to SH was higher than the other direction. The CDD between CD and SH was relatively low in the other years.
4. The CDD between Beijing (BJ) and Shanghai (SH) was relatively high during 2014, 2015, and 2017, in which the CDD from BJ to SH was higher in 2014, but the CDD from SH to BJ was higher in 2015 and 2017.
5. The CDD between Beijing (BJ) and Guangzhou (GZ) was relatively high in 2014, 2016, and 2017, in which the CDD from GZ to BJ was higher in 2014, but the CDD from BJ to GZ became higher from 2015 to 2017.

The findings described above are overall consistent with the results obtained from the correlation coefficients in Table 2. Contrary to the correlation coefficients, however, the CDDs could infer directionality between each pair of cities and its structure change over time.

## 5 Discussion and conclusions

In this work, we presented a statistical measure of directional dependence which is called copula directional dependence (CDD). We obtained the PM2.5 concentration level data for the four major Chinese cities, Beijing, Chengdu, Guangzhou, and Shanghai, from January 1, 2013 to June 30, 2017. We preprocessed the data by using feedforward neural networks with lagged inputs, and we statistically analyzed directional dependence between the cities based on the proposed CDDs. An R source code for preprocessing and analyzing the PM2.5 data in this study is available at S1 Appendix.

Particulate matter consisting of PM2.5 can be generated from various sources, and its concentration can be changed by numerous factors such as wind direction, wind speed, seasonal change, weather, and industrialization [8, 18]. On the other hand, the proposed CDD can infer dependence structures between the major Chinese cities conveniently without requiring complicated models involving the diverse economic or environmental factors in a meteorological system. The inferred CDD structure can characterize economic and environmental relationships between Chinese cities, which may indirectly reflect interrelationships between underlying hidden factor variables.

In this study, we applied CDD using bivariate copulas. A multivariate generalization can be computationally challenging due to the large number of combinations of the variables. Kim and Jung [74] extended the bivariate copula-based directional dependence to a multivariate version by combining it with time-varying partial correlations. It is necessary to develop computationally efficient methods further in future works.

Based on the directional dependence structures obtained by the CDDs, we could visually present the interactions between the four Chinese cities over different time periods. Moreover, we could provide levels of confidence on the directionality of the dependences between the cities by computing bootstrap confidence intervals.

We remark that the CDD values need to be interpreted with caution. The CDD values range between 0 and 1, and a larger value implies a stronger correlation between the variables. However, since the CDD is a version of the coefficient of determination in multiple linear regression according to the definition in Eq (3), it should be expressed with higher precision, e.g., in three to four digits as in Table 4, than the Pearson and Spearman correlation coefficients. Note that the methodology presented in this paper consists of several statistical methods such as copulas, beta regression, and ANNs, each of which can be a source of uncertainty that affects the CDD values. Therefore, statistical decisions made by the bootstrap confidence interval are not always reliable, especially when the CDD values are relatively small. An alternative method for a reliable decision is to introduce a multiple comparision procedure to determine significant nonzero CDD values; see, e.g., [75, 76].

It is remarkable that we could see a structural change in the CDD networks, especially in the directions of the edges of the networks, between the period 2013-2014 and the period 2015-2017. Such a structural change indicates a certain dramatic shift in the states of the underlying latent factors affecting PM2.5 levels around 2014. Trends in air quality in Chinese major cities were analyzed in [17] and it was found that Beijing experienced decreased PM2.5 from 2013 to 2015. Overall, Beijing, Chengdu, and Guangzhou experienced improvements in air quality in the recent decade. Note that China released an ambient air quality standard, GB 3095-2012, in 2012 [14], and key cities including the four major cities analyzed in this study were required to implement the standard. In September 2013, the State Council of China issued the Air Pollution Prevention and Control Action Plan (APPCAP). Implementation of the APPCAP led to a control of ambient air pollution and substantial reductions in mortality and years-of-life-lost (YLLs) [77]. We can guess that continuous efforts in China have resulted in dramatic changes in air quality and the constituents of air pollutants. Further studies on the nature of the changes in air quality are necessary in order to make better public health policies. The proposed CDD can be effectively applied to any further analysis in general settings or for a more detailed analysis of air pollution.

## Supporting information

S1 Appendix Source code. An R source code for preprocessing and analyzing the PM2.5 data in this study.
(PDF)
