# Supplementary material for: Directional dependence between major cities in China based on copula regression on air pollution measurements
Source: PLoS One. 2019 Mar 14;14(3):e0213148. doi: 10.1371/journal.pone.0213148 (PMC6417661; doi:10.1371/journal.pone.0213148)
Supplement: S1 Appendix — An R source code for preprocessing and analyzing the PM2.5 data in this study. (PDF) [file pone.0213148.s001.pdf]

Supporting Information

for the paper "Directional dependence between major cities in China based on copula regression on air pollution measurements"

Jong-Min Kim[1], Namgil Lee[2*], Xingyao Xiao[3]

1 Statistics Discipline, Division of Sciences and Mathematics, University of Minnesota-Morris, Morris, MN 56267-2134, USA
2 Department of Information Statistics, Kangwon National University, Chuncheon, Gangwon 24341, South Korea
3 Applied Statistics and Psychometrics, Lynch School of Education, Boston College, Chestnut Hill, MA 02467, USA

* namgil.lee@kangwon.ac.kr

S1 Appendix. Source code. An R source code for preprocessing and analyzing the PM2.5 data in this study.

```
library(forecast)
library(readxl)
library(lmtest)
library(ggplot2)
library(copula)
library(gcmr)
set.seed(100)
cityabb <- c('BJ', 'CD', 'GZ', 'SH')
data_start <- as.Date("2013-01-01")
data_end <- as.Date("2017-06-30")
#### Select the year ####
year <- (2013:2017)[1]
citylist <- c('Beijing', 'Chengdu', 'Guangzhou', 'Shanghai')
datalist <- vector("list", 4)
for (i in 1:4) {
  #### Select the city ####
  cat("======== Year: ", year, ", City: ", citylist[i], "========\n")
  city <- citylist[i]
  ## Read air quality data files
  ## Be sure that the files are saved in the working directory
  fnreg <- paste0(city, '_', year, '_HourlyPM*') ## file name pattern
  fnlst <- list.files(path = ".", pattern = fnreg, full.names = TRUE,
                      ignore.case = TRUE)
  if(file.exists(fname <- fnlst[1])) {
    datalist[[i]] <- read.csv(fname, header = T, skip = 3)
  } else {
    cat('File does not exist: ', fname, '\n')
  }
  ## Remove missing values & Replace negatives with zero
  ## Original data have no missing values
```

```
  datalist[[i]] <- subset(datalist[[i]], !is.na(Value))
  datalist[[i]]$Value[datalist[[i]]$Value < 0] <- 0
  ## Visualization for hourly PM2.5 data
  Y <- datalist[[i]]$Value
  print(summary(Y))
  print(paste("IQR:", IQR(Y)))
  # Time plot
  plot(Y, type='p', pch=16,
       main=paste0("Time Plot of ",city," PM2.5 Level (",year," Year)"),
       xlab="Hourly", ylab="PM2.5", cex.lab=1.3, cex.main=1.5)
  grid()
  # Histogram
  hist(Y, prob=TRUE,
       main=paste0("Histogram of ",city," PM2.5 Level (",year," Year)"),
       xlim = c(0,600), breaks = seq(0,900,50),
       ylim = c(0,0.02), xlab="PM2.5", cex.lab=1.5, cex.main=1.4)
  lines(density(Y))             # add a density estimate with defaults
  lines(density(Y, adjust=2), lty="dotted") # "smoother" density
}
## Compute daily maximum of PM2.5 levels
datamaxlist <- list('vector', 4)
for (i in 1:4) {
  datamaxlist[[i]] <- with(datalist[[i]],
                          aggregate(Value, list(Month, Day), max))
  colnames(datamaxlist[[i]]) <- c("Month", "Day", cityabb[i])
}
## Merge daily maximum data sets by "Month" and "Day"
chinadata0 <- datamaxlist[[1]]
for (i in 2:4) {
  chinadata0 <- merge(chinadata0, datamaxlist[[i]], sort = FALSE)
}
chinadata1 <- chinadata0[with(chinadata0, order(Month, Day)), -c(1,2)]
## Remove negatives(which have been replaced with zeros)
chinadata <- chinadata1[apply(chinadata1 > 0, 1, all),]
## Print correlation
sp_cor <- cor(chinadata, use = "complete.obs", method = "spearman")
print('Spearman correlation coefficients:')
print(round(sp_cor, 2))
## Neural Network Autoregression
ResCities <- vector('list', 4)
for (idcity in 1:4) {
  fit_cy <- nnetar(chinadata[,idcity])
  fcast_cy <- forecast(fit_cy)
  plot(fit_cy$fitted, fit_cy$residuals, xlab='Fitted', ylab='Residual',
       cex.lab = '1.5', main = paste('Forecasts from', fcast_cy$method))
  plot(chinadata[,idcity], type='l', lty=2, col='red', ylab='PM2.5',
       main = paste('Forecasts from', fcast_cy$method),
       cex.main = 1.5, cex.lab = 1.5)
  lines(fit_cy$fitted, lwd = 1.5)
  legend('topright', legend = c('Observed', 'Fitted'),
         lty=c(2,1), col=c('red','black'), lwd=c(1,1.5), cex=1.5)
  ## Durbin-Watson test for autocorrelation
```

```
  print(dwtest(y~., data = data.frame(y = fit_cy$residuals[-(1:fit_cy$p)])))
  ## Compute residuals
  ResCities[[idcity]] <- fit_cy$residuals[-(1:17)]
}
## Select two cities and compute COPULA DIRECTIONAL DEPENDENCE
idcity1 = 1; idcity2 = 2
data <- data.frame(u = ResCities[[idcity1]], v = ResCities[[idcity2]])
## Transform data to uniform d.f. through its empirical d.f.
Empiric.df<-function(data,x) {
  data<-sort(data)
  if(min(data)>0) a<-0 else a<-floor(min(data)/100)*100
  if(max(data)<0) b<-0 else b<-ceiling(max(data)/100)*100

  for(j in 1:length(x)) {
  if(x[j]<a) x[j]<-a
  if(x[j]>b) x[j]<-b
  }
  data<-c(a,data,b)
  n<-length(data)
  p<-c(rep(0,(n-1)))
  q<-c(rep(0,(n-1)))

  for(i in 2:(n-2)) {
  p[i]<-(data[i]+data[i+1])/2
  q[i]<-(i-1)/(n-2)
  }
  p[1]<-a
  p[n-1]<-b
  q[1]<-0
  q[n-1]<-1
  approx(p,q,xout=c(x))$y
}
## Transform original data to U(0,1)
Emp.index <- data.frame(u = Empiric.df(data[,1], data[,1]),
                        v = Empiric.df(data[,2],data[,2]))
## Compute CDD
r12 <- gcmr(u~v, data = Emp.index, marginal = beta.marg(link = "logit"),
            cormat = arma.cormat(0, 0) )
Er12 <- exp(r12$estimate[1] + Emp.index$v * r12$estimate[2])/
            (1 + exp(r12$estimate[1] + Emp.index$v * r12$estimate[2]))
vtou_rho2 <- var(Er12)/var(Emp.index$u)
r21 <- gcmr(v~u, data = Emp.index, marginal = beta.marg(link = "logit"),
            cormat = arma.cormat(0, 0) )
Er21 <- exp(r21$estimate[1] + Emp.index$u * r21$estimate[2])/
            (1 + exp(r21$estimate[1] + Emp.index$u * r21$estimate[2]))
utov_rho2 <- var(Er21)/var(Emp.index$v)
rslt <- data.frame(vtou_rho2,utov_rho2)
## Step to compute Skewness adn Kurtosis
dat.man <- matrix(rep(0,2000),1000,2)
for (k in 1:1000) {
  n <- 150
  data <- as.matrix(Emp.index[sample(nrow(Emp.index), n), ])
```

```
  dat <- data.frame(data)
  m11 <- gcmr(u~v, data = dat, marginal = beta.marg(link = "logit"),
  cormat = arma.cormat(0, 0))
  Er12 <- exp(m11$estimate[1] + dat$v*m11$estimate[2])/
            (1+exp(m11$estimate[1]+dat$v*m11$estimate[2]))
  dat.man[k,1] <- var(Er12)/var(dat$u)
  m41 <- gcmr(v~u, data = dat, marginal = beta.marg(link = "logit"),
  cormat = arma.cormat(0, 0))
  Er21 <- exp(m41$estimate[1] + dat$u * m41$estimate[2])/
            (1 + exp(m41$estimate[1] + dat$u * m41$estimate[2]))
  dat.man[k,2] <- var(Er21)/var(dat$v)
}
data.direction <- as.data.frame(dat.man)
colnames(data.direction) <- c("vtou_rho2", "utov_rho2")
head(data.direction)
##
B <- 99999
d <- data.direction$vtou_rho2 - data.direction$utov_rho2
m0 <- mean(d)
rndmdist <- replicate(B, mean((rbinom(length(d), 1, .5)*2 - 1) * d))
sum(abs(rndmdist) > abs(m0))/length(rndmdist) # gives p =  4.00004e-05
## confidence intervals
library("boot")
mean.fun <- function(dat, idx) mean(dat[idx], na.rm = TRUE)
boot.out <- boot(d, mean.fun, R=3000)
boot.out
boot.ci(boot.out)
```
